# Fiber Type-Specific Satellite Cell Content in Cyclists Following Heavy Training with Carbohydrate and Carbohydrate-Protein Supplementation

**DOI:** 10.3389/fphys.2016.00550

**Published:** 2016-11-16

**Authors:** Alec I. McKenzie, Andrew C. D'Lugos, Michael J. Saunders, Keith D. Gworek, Nicholas D. Luden

**Affiliations:** Human Performance Laboratory, James Madison UniversityHarrisonburg, VA, USA

**Keywords:** endurance-trained cyclists, intensified training, myogenesis, protein supplementation

## Abstract

The central purpose of this study was to evaluate the fiber type-specific satellite cell and myonuclear responses of endurance-trained cyclists to a block of intensified training, when supplementing with carbohydrate (CHO) vs. carbohydrate-protein (PRO). In a crossover design, endurance-trained cyclists (*n* = 8) performed two consecutive training periods, once supplementing with CHO (de facto “control” condition) and the other with PRO. Each training period consisted of 10 days of intensified cycle training (ICT–120% increase in average training duration) followed by 10 days of recovery (RVT–reduced volume training; 33% volume reduction vs. normal training). Skeletal muscle biopsies were obtained from the vastus lateralis before and after ICT and again following RVT. Immunofluorescent microscopy was used to quantify SCs (Pax7+), myonuclei (DAPI+), and myosin heavy chain I (MyHC I). Data are expressed as percent change ± 90% confidence limits. The 10-day block of ICT_CHO_ increased MyHC I SC content (35 ± 28%) and myonuclear density (16 ± 6%), which remained elevated following RVT_CHO_ (SC = 69 ± 50% vs. PRE; Nuclei = 17 ± 15% vs. PRE). MyHC II SC and myonuclei were not different following ICT_CHO_, but were higher following RVT_CHO_ (SC = +33 ± 31% vs. PRE; Nuclei = 15 ± 14% vs. PRE), indicating a delayed response compared to MyHC I fibers. The MyHC I SC pool increased following ICT_PRO_ (37 ± 37%), but without a concomitant increase in myonuclei. There were no changes in MyHC II SC or myonuclei following ICT_PRO_. Collectively, these trained endurance cyclists possessed a relatively large pool of SCs that facilitated rapid (MyHC I) and delayed (MyHC II) satellite cell proliferation and myonuclear accretion under carbohydrate conditions. The current findings strengthen the growing body of evidence demonstrating alterations in satellite cell number in the absence of hypertrophy. Satellite cell pool expansion is typically viewed as an advantageous response to exercise. However, when coupled with our previous report that PRO possibly enhanced whole muscle recovery and increased MyHC I and II fiber size, the limited satellite cell/myonuclear response observed with carbohydrate-protein seem to indicate that protein supplementation may have minimized the necessity for satellite cell involvement, thereby suggesting that protein may benefit skeletal muscle during periods of heavy training.

## Introduction

The central dogma of muscle regeneration and stress adaptation involves a subset of multipotent skeletal muscle stem cells' commitment to a cell lineage involving satellite cell proliferation, differentiation, and eventual fusion to target muscle fibers in the form of myonuclei of myotubes. This process is critical for healthy muscle regeneration and development. Most of what is currently known about human satellite cell behavior in the context of exercise has been derived from resistance exercise models. It is now widely accepted that resistance exercise is a powerful stimulus for satellite cell activation and proliferation (Crameri et al., 2004, 2007; O'Reilly et al., 2008; Mikkelsen et al., 2009; Babcock et al., 2012; Cermak et al., 2013) and, though subject to debate (O'Connor and Pavlath, 2007; McCarthy et al., 2011), satellite cell differentiation is probably permissive for large-scale muscle growth (Rosenblatt et al., 1994; Barton-Davis et al., 1999; Li et al., 2006; Petrella et al., 2008). While less is known about satellite cell behavior in response to endurance exercise, there is growing evidence that heavy endurance exercise can stimulate satellite cell proliferation following as little as one session (Darr and Schultz, 1987; Mackey et al., 2007) and that aerobic training programs can result in measurable increases in satellite cell number (Umnova and Seene, 1991; Charifi et al., 2003; Li et al., 2006; Verney et al., 2008; Shefer et al., 2010; Kurosaka et al., 2012; Fry et al., 2014; Hoedt et al., 2016; Murach et al., 2016). Notably, satellite cell pool expansion has been documented following aerobic training not only with- (Umnova and Seene, 1991; Charifi et al., 2003; Verney et al., 2008; Shefer et al., 2010; Fry et al., 2014; Murach et al., 2016) but also without measurable hypertrophy (Li et al., 2006; Kurosaka et al., 2012; Hoedt et al., 2016). Also, chronic low-frequency stimulation (akin to endurance exercise) of animal muscle and high-intensity interval cycle training in humans induces satellite cell proliferation in the absence of muscle hypertrophy (Putman et al., 2001; Joanisse et al., 2013). Further, 6 weeks of cycle training, regardless of training intensity, led to an increase in satellite cell activity (Joanisse et al., 2015), altogether supporting the idea that satellite cells have a distinct role in non-hypertrophic remodeling.

All of the aforementioned training studies were performed with previously untrained subjects. As such, nothing is known about the satellite cell behavior of trained muscle exposed to an abbreviated block of heavy exercise; a common training strategy practiced by endurance athletes. We had the unique opportunity to examine muscle tissue obtained from a larger investigation of trained cyclists that underwent two separate phases of intensified training and recovery, with and without protein supplementation (D'Lugos et al., 2016). Therefore, our primary aim was to quantify fiber-type specific satellite cells (Pax7+) and myonuclei before and after 10 days of intensified training and again following 10 days of subsequent recovery.

The potential for protein intake to augment the satellite cell response to resistance exercise has also recently been investigated. Specifically, several groups reported that whey protein supplementation stimulates a greater satellite cell response to resistance exercise training compared to placebo (Olsen et al., 2006; Farup et al., 2014), though others reported null findings when evaluated after a single exercise session (Roberts et al., 2010; Snijders et al., 2014). Because the principal investigation was designed to test the physiological effects of carbohydrate-protein supplementation proximal to each training session, we were able to detail the impact of carbohydrate-protein supplementation, compared to carbohydrate-only, on satellite cells within an endurance-training model. The secondary aim was therefore to compare fiber-type specific satellite cell responses with carbohydrate-protein supplementation proximal to each training session to carbohydrate-only. Collectively, these data expand our understanding of the diverse nature of satellite cells, which is important for the development of interventions designed to optimize skeletal muscle health and performance.

## Methods

### Study design

This project was ancillary to a larger study designed to assess the effect of carbohydrate-protein supplementation on performance and physiological tolerance to heavy aerobic cycling training (D'Lugos et al., 2016). Ten endurance-trained cyclists enrolled in the study. However, one subject dropped out of the study prior to completion citing general intolerance to the training overload and another subject was dismissed for non-compliance with standardization procedures. Subject demographics from the remaining 8 subjects (6 males; 2 females) are displayed in Table 1. All subjects possessed a VO_2max_ of ≥ 50 ml·kg^−1^·min^−1^, and completed ≥ 7 h of cycling per week with ≥ 1 long ride (≥ 3 h) per week for ≥ 2 months prior to beginning the study. Before any testing, subjects were provided with written and oral details about the experimental procedures and associated risks. Study procedures were approved by the James Madison University Institutional Review Board.

**Table 1:** **Subject demographics**.

	**Age (yrs)**	**VO_2max (ml·kg^−1^·min^−1^)_**	**Peak watts**	**Height (cm)**	**Body mass (kg)**
Subjects (*n* = 8)	25 ± 7	63.1 ± 8.4	319 ± 55	157 ± 11	72 ± 12

Data are expressed as means ± SD. Peak Watts – Power output during the final complete stage of VO_2max_ test.

### General experimental design

**Figure 1:**
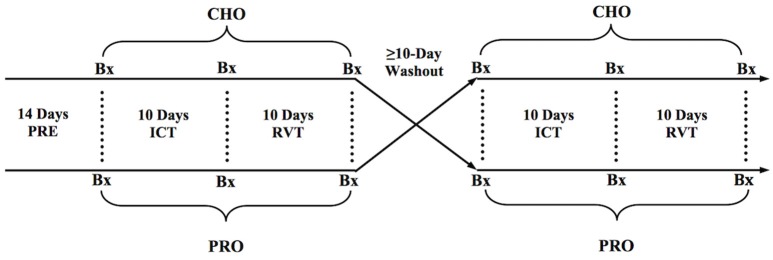
**General experimental design**. Bx, Skeletal muscle biopsy.

To examine the impact of carbohydrate (CHO) and carbohydrate-protein (PRO) supplementation during and following intensified training, subjects performed two identical blocks of training separated by a ≥ 10-day washout period. In a counterbalanced randomized fashion, subjects were provided with CHO or PRO throughout each training block. Each training block consisted of normal training (PRE), intensified cycle training (ICT), and reduced-volume training (RVT) (Figure 1). It is worth noting that the 10-day washout lead to ≥ 27 days between the two ICT phases, as the washout period was preceded by 10 days of RVT and followed by 7 days of NT prior to the subsequent ICT phase. While the time lapse between ICT phases was sufficient to “washout” the impact of the initial training block (and nutrition) on cycling performance, cardiovascular function, and skeletal muscle function, the amount of time necessary for true washout of CSA, satellite cell density, and myonuclear density is not known. However, there were no clear statistical differences between PRE_CHO_ and PRE_PRO_ CSA, satellite cell density, or myonuclear density.

### Training

Complete training data was previously reported (D'Lugos et al., 2016). Briefly, mean (± *SD*) training duration, power output, and heart rate during PRE were: CHO− 62 ± 8 min, 190 ± 29 W, and 150 ± 15 bpm; PRO- 63 ± 6 min, 183 ± 28 W, and 145 ± 13 bpm. Training data during ICT were: CHO− 137 ± 15 min, 169 ± 32 W, and 139 ± 12 bpm; PRO− 140 ± 17 min, 171 ± 27 W, and 137 ± 12 bpm. Training data during RVT were: CHO− 41 ± 5 min, 198 ± 37 W, 151 ± 12 bpm; PRO− 42 ± 5 min, 198 ± 36 W, and 147 ± 11 bpm.

#### Normal training (PRE)

Participants were provided with a heart rate monitor and rear bicycle wheel equipped with an integrated PowerTap system (Saris Cycling Group Inc, Madison WI) to allow us to quantify cycling workload outside of the laboratory. Prior to the intervention, subjects were instructed to perform customary training habits for 2 weeks. Training during this period was used to prescribe training volumes and intensities throughout the investigation.

#### Intensified cycling training (ICT)

Immediately following PRE, subjects performed 10 days of ICT, consisting of a 120% increase in average daily training volume. This training load was intended to simulate a typical “training camp” experience, and was modeled after a design used by Witard et al. (2011). Cycling performance trials (~3 h each) were completed in the laboratory on days 1, 4, 7, and 10 and contributed to the total training load during ICT. Training sessions on days 2, 3, 5, 6, 8, and 9 were performed outside of the laboratory. Subjects were provided with detailed individualized training guidelines for these days, based on their training volume during PRE. Power output, heart rate, and training duration were recorded to verify compliance with the training guidelines.

#### Reduced volume training (RVT)

Immediately following ICT, subjects performed 10 days of RVT where average daily training volume was reduced to ~60% of PRE and ~30% of ICT. Similar to above, power output, heart rate, and training duration was recorded during all training sessions.

### Nutritional treatments and dietary controls

Treatment beverages were administered during and immediately following each training session throughout ICT and RVT. During lab trials, subjects ingested 250 ml of fluid every 20 min until completion (750 ml·h^−1^; details provided below). For all rides performed in the field (ICT 2, 3, 5, 6, 8, 9, and RVT days 2–9), participants consumed 500 ml of fluid during each 40-min segment (750 ml·h^−1^). Following each ride, participants consumed an individualized volume of fluid within 30 min of terminating exercise. Participants were instructed to abstain from any other beverage or food intake for 2 h following the completion of each exercise session, with the exception of *ad libitum* water consumption. Additionally, subjects were instructed to record any volume of unfinished post-exercise beverages. Treatment details were previously described (D'Lugos et al., 2016). Briefly, for the carbohydrate intervention, during-exercise drinks consisted of Gatorade® (6% CHO by volume) delivered at 45 g CHO·h^−1^ whereas post-exercise carbohydrate drinks consisted of a mixture of chocolate flavored energy gels (Clif Shots) that delivered 1.2 g CHO·kg BW^−1^. For the carbohydrate-protein intervention, during-exercise drinks consisted of Gatorade® with additional hydrolyzed whey protein isolate (AMCO, Burlington NJ) (6% carbohydrate+2% protein by volume) delivered at 45 g CHO·h^−1^ and 15 g PRO·h^−1^. Post-exercise carbohydrate-protein drinks consisted of non-fat chocolate milk. Each serving consisted of 9.93 ml·kg BW^−1^ and provided 1.2 g CHO·kg BW^−1^ and 0.4 g PRO·kg BW^−1^.

#### Dietary controls

A thorough description of the dietary procedures has been previously described (D'Lugos et al., 2016). Dietary intake was also recorded throughout ICT (10 days) and RVT (10 days). Using copies of dietary records obtained from the first training period, subjects were instructed to replicate their dietary habits during the second period of training. During the second period, subjects were provided with forms listing their diet from the first phase. All laboratory tests (i.e., skeletal muscle biopsies, VO_2max_ tests, and TT) were performed after an 8–10 h overnight fast (*ad libitum* water consumption). In addition, subjects were provided with a standardized boxed-lunch (James Madison University Dining Services–ARAMARK) following all laboratory sessions. Subjects were to consume all contents of the boxed-lunch during the 2–6-h post-exercise period with only *ad libitum* water consumption permitted during this time period. This allowed for the standardization of dietary intake for approximately 6 h after each laboratory session.

### Dependent measures

#### VO_2max_ tests

Subjects performed an initial incremental exercise test on a computerized cycle ergometer (Velotron, Racermate, Seattle WA). Subjects were instructed to select a workload that they could comfortably sustain for approximately 1 h of cycling. Starting with their self-selected intensity, subjects performed continuous 2-min stages with 25-Watt workload increments until volitional exhaustion or subjects could no longer maintain a cadence of 50 rpm. Oxygen uptake (VO_2_) was assessed at each stage in 30-s intervals using indirect calorimetry via an automated Moxus Modular Metabolic System (AEI Technologies, Bostrop, TX).

#### Skeletal muscle biopsies

Skeletal muscle biopsies were obtained from the *vastus lateralis* (VL) 3 days before the onset of ICT, on the first day of RVT, and again 1 day following RVT for both treatments (six total biopsies). All biopsies were obtained in the morning hours with no more than 60 min of variation between any two biopsies for a given subject. Immediately following each biopsy, samples were dried of excess blood and any visible adipose/connective tissue was removed. Samples (~100 mg) were mounted in tragacanth gum, immersed in liquid nitrogen-cooled isopentane and stored at −80°C until analysis. Ten micrometer serial cross sections were cut and mounted onto slides at −25°C (Minotome Plus; Triangle Biomedical Sciences, Durham, NC). Due to insufficient tissue yield from 2 subjects during RVT (PRO), this time-point was excluded from statistical analysis.

#### Immunohistochemistry: Pax7, laminin, MyHC I

Skeletal muscle satellite cell content was quantified using traditional immunohistochemical methods adapted from Fry et al. (2016). Day 1: Slides were fixed in 4% paraformaldehyde for 7 mins then subsequently underwent epitope retrieval protocol using sodium citrate (10 mM, pH 6.5) at 92°C. Slides were washed in PBS and incubated overnight at 4°C in primary antibodies directed against myosin heavy chain 1 (BA.D5-C, 1:75, Developmental Studies Hybridoma Bank, Iowa City, Iowa) and laminin (no. L9393, 1:200, Sigma-Aldrich). Day 2: Following a wash in PBS, endogenous peroxidase activity was blocked with a 7-min wash in 3% H_2_O_2_. Slides were then washed in PBS and incubated for 1 h with appropriate secondary antibodies (MyHC I: Goat anti-mouse IgG2b, Alexa Fluor 647, 1:250, Invitrogen A21242; Laminin: Goat anti-rabbit IgG1, Alexa Fluor 555, 1:500, Invitrogen A21429). Next, slides were washed in PBS and subsequently blocked in 2.5% normal horse serum (NHS) (Vector, S-2012) for 1 h at room temperature, followed by an overnight incubation (4°C) with Pax7 primary antibody (Pax7-C, 1:100, Developmental Studies Hybridoma Bank, Iowa City, Iowa). Day 3: Slides were then incubated for 1 h at room temperature in goat anti-mouse biotinylated secondary antibody (Cat# 115-065-205, 1:1000 in 2.5% NHS, Jackson Immuno Research, West Grove, PA). Utilizing a tyramide signal amplification (TSA) kit (T20932, Life Technologies), slides were incubated for 1 h in streptavidin-horseradish peroxidase (1:100), and then for 20 min in AF488 tyramide (1:200). Lastly slides were counterstained with DAPI (#D35471, 1:10000, Invitrogen), washed in PBS, and mounted with fluorescent mounting media (H-1000, Vector).

#### Imaging and quantification

**Figure 2:**
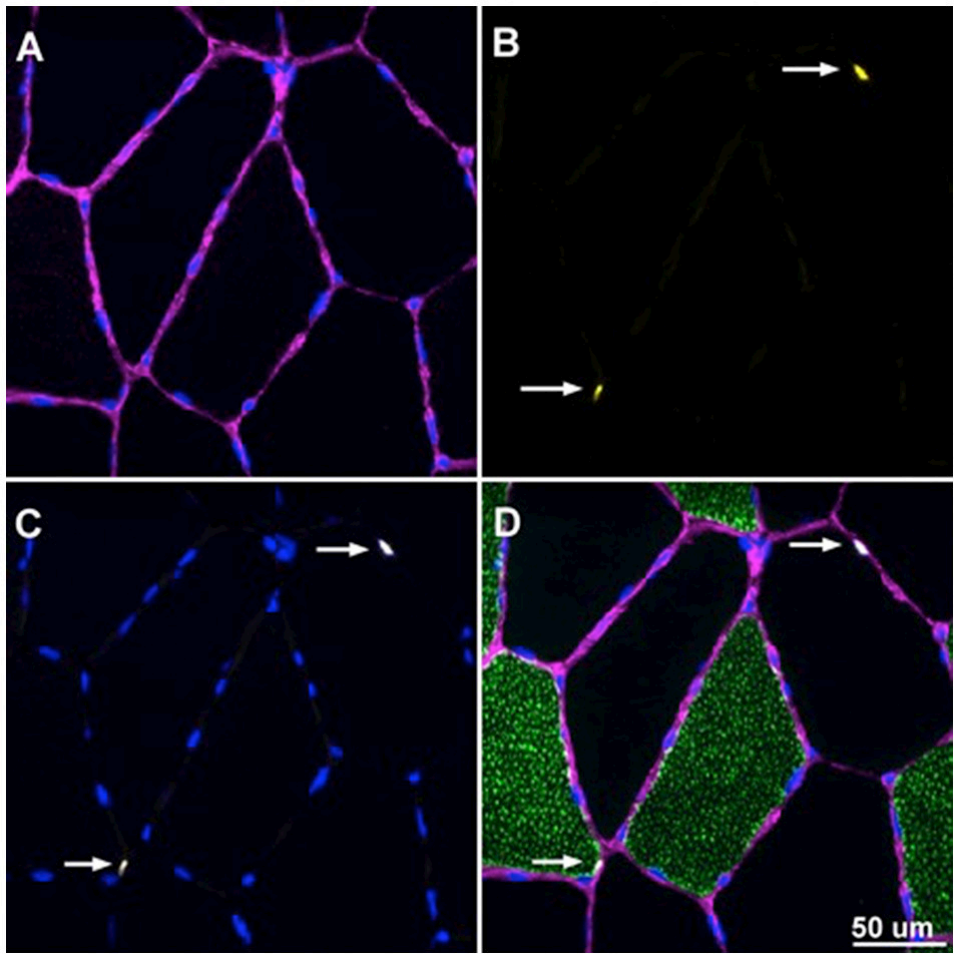
**Overlaid image of Pax7+/DAPI+ cells enclosed by laminin border**. Image was captured at 20X magnification. **(A)** Laminin border of skeletal muscle fibers appearing purple with DAPI+ cells appearing blue. **(B)** Pax7+ structures appearing yellow denoted with white arrows. **(C)** DAPI+ cells appearing blue with white arrows denoting the myonuclei of Pax7+/DAPI+ cells. **(D)** Overlaid image with laminin, Pax7, and DAPI with white arrows denoting SCs that are Pax7+/DAPI+ and enclosed by a laminin border.

Slides were analyzed, with the investigator blinded from both time-point and treatment phase, using an upright fluorescent microscope (Nikon Eclipse Ti-E inverted microscope equipped with an automated stage, Tokyo Japan). Samples analyzed for satellite cell content were imaged at 20X magnification and subsequently merged to seamlessly visualize the cross section in its entirety. Objects that were Pax7+/DAPI+ within a laminin border were counted as satellite cells. Myonuclei were assessed by counting objects stained DAPI+ that were also under the laminin border. Objects that were Pax7+/DAPI+ were not included in the myonuclear count. Captured images in each filter block were overlaid using NIS Elements software (Nikon, Tokyo Japan). MyHC I, Pax7+/DAPI+ cell counting, fiber cross sectional area (CSA μm^2^) and fiber nuclear content were determined using ImageJ software (U.S. National Institutes of Health, Bethesda, Maryland). A sample image is displayed in Figure 2. Note that fiber CSA from 7 of the 8 subjects in the current study was previously reported (D'Lugos et al., 2016).

#### Statistical analysis

All data was log transformed to diminish the effects of non-uniformity. Magnitude-based inferences about the data were derived using methods described by Hopkins (2004). The threshold for the smallest meaningful effect was determined by 0.2 x within subject *SD* obtained from data gathered from the PRE biopsy tissue under CHO conditions. A published spreadsheet (Hopkins, 2007) was used to determine the likelihood of the true treatment effect (of the population) reaching the substantial change threshold (0.2 *SD*); these are classified as <1% almost certainly no chance, 1–5% = very unlikely, 5–25% = unlikely, 25–75% = possible, 75–95% = likely, 95–99% = very likely, and >99% = almost certain. If the percent chance of the effect reaching the substantial change threshold was <25% and the effect was clear, it was classified as a “trivial” effect. If 90% confidence intervals included values that exceeded the substantial change threshold for both a positive and negative effect, effects were classified as unclear (>5% chance of reaching the substantial threshold for both a positive and negative effect). For ease of interpretation, *P*-values derived from simple contrasts between time-points are included alongside the magnitude-based inferential outcomes. Data are displayed as raw means ± *SD* and/or percent difference between time points ± CL (90% confidence limit; to illustrate uncertainty in treatment effects).

## Results

### Muscle sample profile

An average of 478 ± 281 fibers were included in the satellite cell analysis of each sample; 222 ± 148 MyHC I fibers and 255 ± 160 MyHC II fibers were analyzed at each time point. An average of 155 ± 60 fibers were included in the analysis of myonuclei (total and centrally located myonuclei); 74 ± 39 MyHC I fibers and 78 ± 38 MyHC II fibers were analyzed at each time point.

### Satellite cell density (satellite cells/muscle 100 fibers)

CHO and PRO SC data are presented in Table 2. Data are expressed as percent change ± 90% confidence limits. Pooled SC density was *Likely* higher (22 ± 28%) following ICT_CHO_ compared to PRE_CHO_ and remained *Most Likely* higher (44 ± 24%) than PRE_CHO_ following RVT_CHO_. MyHC I SC density *Very Likely* increased (35 ± 28%) from PRE_CHO_ to ICT_CHO_, and again *Likely* increased (20 ± 26%) from ICT_CHO_ to RVT_CHO_ Therefore, MyHC I SC density was *Very Likely* elevated (69 ± 50%) following RVT_CHO_ compared to PRE_CHO_. MyHC II SC density was similar between PRE_CHO_ and ICT_CHO_, but was *Likely* higher (33 ± 31%) following RVT_CHO_ compared to PRE_CHO_. ICT_PRO_ led to a *Very Likely* increase (37 ± 37%) in MyHC I SC density compared to PRE_PRO_, though MyHC II SC density was unchanged under PRO conditions. This translated to a *Likely* increase (18 ± 27%) in pooled SC density from PRE_PRO_ to ICT_PRO_. There were no clear differences in the response to ICT (PRE vs. ICT) between CHO and PRO. As mentioned in the methods, due to insufficient tissue yield from 2 subjects during RVT_PRO_, this time-point was excluded from statistical analysis. However, among the 6 subjects, SC density appeared to be similar between ICT_PRO_ and RVT_PRO_, for pooled (14.4 vs. 13.7 SC/100 fibers for ICT and RVT, respectively), MyHC I (12.9 vs. 10.8 SC/100 fibers), and MyHC II fibers (16.1 vs. 16.4 SC/100 fibers).

**Table 2:** **Satellite cell density (SCs/100 Fibers)**.

	**Carbohydrate**			**Protein**	
	**PRE**	**ICT**	**RVT**	**PRE**	**ICT**
Pooled SC density	13.4 ± 4.3	16.2 ± 4.8^a^	19.6 ± 7.5^b^	12.5 ± 5.8	14.5 ± 5.5^c^
MyHC I SC density	10.7 ± 3.0	14.6 ± 5.0^d^, ^i^	18.5 ± 6.7^e^, ^f^	10.7 ± 4.9	14.1 ± 4.4^h^
MyHC II SC density	15.3 ± 6.7	17.8 ± 7.0	21.4 ± 10.3^g^	13.6 ± 6.3	15.8 ± 8.4

*Data are expressed as means ± SD. LH, Likelihood*.

a *Likely different vs. PRE (86% LH; p = 0.13)*;

b *Most Likely different vs. PRE (100% LH; p = 0.003)*;

c *Likely different vs. PRE (80% LH; p = 0.21)*;

d *Very Likely different vs. PRE (97% LH; p = 0.03)*;

e *Likely different than ICT (83% LH; p = 0.23)*;

f *Very Likely different vs. PRE (99% LH; p = 0.01)*;

g *Likely different vs. PRE (93% LH; p = 0.05)*;

h *Very Likely different vs. PRE (95% LH; p = 0.06)*;

i *Likely different response from PRE to ICT vs. PRO (89% LH; p = 0.01)*.

### Myonuclear density (myonuclei/fiber) and centrally located nuclei

CHO and PRO myonuclear data are presented in Table 3. Data are expressed as percent change ± 90% confidence limits. Pooled nuclear density was *Possibly* higher (6 ± 9%) following ICT_CHO_ compared to PRE_CHO_ and was *Likely* higher (13 ± 13%) than PRE_CHO_ following RVT_CHO_. MyHC I nuclear density *Likely* increased (8 ± 8%) from PRE_CHO_ to ICT_CHO_ and *Very Likely* remained higher (13 ± 8%) following RVT_CHO_ compared to PRE_CHO_. MyHC II nuclear density did not change from PRE_CHO_ to ICT_CHO_, *Possibly* increased (8 ± 11%) from ICT_CHO_ to RVT_CHO_, and was *Likely* higher (14 ± 15%) at RVT_CHO_ compared to PRE_CHO_. No changes in either MyHC I or MyHC II nuclear density were observed with ICT_PRO_. Therefore, there was a treatment effect, as MyHC I nuclear density *Likely* increased to a greater extent following ICT_CHO_ compared to ICT_PRO_. Again, due to insufficient tissue yield from 2 subjects during RVT_PRO_, this time-point was excluded from statistical analysis. For the data available from 6 subjects, pooled nuclear density was 3.35 (ICT_PRO_) and 3.02 nuclei/fiber (RVT_PRO_), MyHC I nuclear density was 3.17 and 2.92 nuclei/fiber, and MyHC II nuclear density was 3.58 and 3.23 nuclei/fiber.

**Table 3:** **Myonuclear density (Myonuclei/Fiber), and myonuclear domain (μm^2^/Nucleus)**.

	**Carbohydrate**			**Protein**	
	**PRE**	**ICT**	**RVT**	**PRE**	**ICT**
Pooled myonuclei	3.3±0.5	3.5±0.8	3.8 ± 0.8^a^	3.4±0.9	3.4±0.9
MyHC I myonuclei	3.2±0.6	3.5 ± 0.8^b^	3.6 ± 0.9^c^	3.3±0.9	3.3±0.8
MyHC II myonuclei	3.5±0.7	3.7±0.9	4.0 ± 1.0^d^	3.5±1.0	3.6±1.0
Pooled myonuclear domain	1671±231	1603±266	1628±389	1479±220	1679 ± 165^e^
MyHC I myonuclear domain	1672±259	1591±266	1658±416	1450±183	1678 ± 209^f^
MyHC II myonuclear domain	1588±204	1556±295	1520±295	1454±290	1635 ± 210^g^

*Data are expressed as means ± SD. Myonuclear domain was calculated using CSA data previously reported (D'Lugos et al., 2016). LH, Likelihood*.

a *Likely different vs. PRE (90% LH; p = 0.08)*;

b *Likely different vs. PRE (84% LH; p = 0.07)*;

c *Very Likely different vs. PRE (96% LH; p = 0.01)*;

d *Likely different vs. PRE (88% LH; p = 0.10)*;

e *Very Likely different vs. PRE (98% LH; p = 0.01)*;

f *Very Likely different vs. PRE (99% LH; p = 0.007)*;

g *Likely different vs. PRE (93% LH; p = 0.03)*.

Though not on a large scale, the percentage of pooled fibers possessing centrally located nuclei *Very Likely* decreased from 1.9 ± 1.21% at PRE_CHO_ to 0.9 ± 0.8% following ICT_CHO_, and then *Very Likely* increased to 3.5 ± 2.6% following RVT_CHO_. This result was driven by MyHC I fibers, as the percentage of MyHC I centrally located nuclei *Very Likely* decreased from 2.6 ± 2.7% at PRE_CHO_ to 0.5 ± 1.0% following ICT_CHO_, and then *Most Likely* increased to 3.5 ± 2.6% following RVT_CHO_. All other comparisons were “unclear.” For the data available from 6 subjects, the percentage of fibers that contained centrally located nuclei were 3.6 (ICT_PRO_) and 1.9% (RVT_PRO_) of pooled fibers, 2.0 and 1.4% of MyHC I fibers, and 4.6 and 1.9% of MyHC II fibers.

### Cross sectional area (μM^2^)

CSA data on MyHC I and MyHC II fibers were previously reported (D'Lugos et al., 2016). Pooled fiber CSA data are presented here. Pooled fiber CSA (means ± *SD*) under CHO conditions were as follows: PRE_CHO_, 5524 ± 1244; ICT_CHO_, 5557 ± 966; and RVT_CHO_, 6055 ± 1592 μm^2^. CSA comparisons across these CHO time points were “unclear.” Pooled fiber CSA under PRO conditions were as follows: PRE_PRO_, 5038 ± 1660; and ICT_PRO_, 5658 ± 1390 μm^2^. Pooled CSA *Likely* increased [percent change ± 90% confidence limits (14 ± 14%)] following ICT compared to PRE. For the data available on 6 subjects, pooled CSA was 5669 μm^2^ (ICT_PRO_) and 5047 μm^2^ (RVT_PRO_), MyHC I CSA was 5295 and 4801 μm^2^ nuclei/fiber, and MyHC II CSA was 5877 and 5266 μm^2^.

### Myonuclear domain (μM^2^/Nucleus)

Myonuclear domains were calculated using previously reported CSA data on MyHC I and MyHC II fibers (D'Lugos et al., 2016). CHO and PRO myonuclear domain data are presented in Table 3. Data are expressed as percent change ± 90% confidence limits. The myonuclear domains of pooled, MyHC I and MyHC II fibers were similar across all CHO time points. Conversely, pooled myonuclear domain *Very Likely* increased (14 ± 9%) from PRE_PRO_ to ICT_PRO_. Likewise, the MyHC I (*Very Likely*; 16 ± 9%) and MyHC II (*Likely*; 13 ± 10%) myonuclear domains increased from PRE_PRO_ to ICT_PRO_. For the data available on 6 subjects, pooled myonuclear domain was 1711 μm^2^/Nucleus (ICT_PRO_) and 1673 μm^2^/Nucleus (RVT_PRO_), MyHC I myonuclear domain was 1701 and 1649 μm^2^/Nucleus, and the MyHC II myonuclear domain was 1647 and 1635 μm^2^/Nucleus.

## Discussion

The goal of this project was to assess the muscle fiber type-specific SC response to 10 days of intensified cycling training in trained cyclists supplementing with carbohydrate or carbohydrate+protein. The most notable findings were the rapid increase in MyHC I satellite cells and myonuclei coupled with a delayed increase in MyHC II satellite cells and myonuclei under CHO conditions. This is the first evidence in humans that chronically cycling-trained skeletal muscle possesses a relatively large satellite cell pool and that a short block of intensified training elicits a fiber type-specific satellite cell/nuclear response. A secondary goal was to assess the impact of PRO supplementation on the satellite cell response to intensified cycling compared to CHO alone. In contrast to CHO supplementation, MyHC I SCs increased following ICT without myonuclear accretion. Furthermore, in these same subjects, MyHC I and II fiber size only increased following intensified exercise with PRO supplementation (D'Lugos et al., 2016), collectively inferring that PRO supplementation may be beneficial for skeletal muscle during periods of heavy endurance training.

Little is known about the SC profile of chronically trained-, particularly endurance-trained skeletal muscle. To our knowledge, SCs have been quantified in endurance-trained athletes on only two other occasions (Mackey et al., 2007; Frese et al., 2016). The satellite cell population observed here, prior to ICT, seem to be in line with previous work, though disparities between subject groups and methodology make it difficult to compare across studies. Satellite cells were assessed in a cohort of runners with aerobic fitness levels similar to the current subjects (~60 ml/kg/min) (Mackey et al., 2007). Unfortunately, raw satellite cell counts were not reported and analysis was not fiber type-specific, but the runners and the current subjects possessed remarkably similar satellite cell levels when expressed relative to myonuclei (runners = 4.1; current subjects = 3.9). More relevant, the myogenic program of elite junior cyclists was recently described and while fiber type-specific satellite cells were again not reported, the combined satellite cell count was ~13 SC/100 fibers (Frese et al., 2016), which is identical to the 13 SC/100 fibers observed here (11 SC/100 MyHC I fibers; 15 SC/100 MyHC II fibers). These numbers, especially the MyHC II population, are moderately higher than the 5–10 SC/100 muscle fibers typically found in untrained subjects (Snijders et al., 2015) and consistent with the 10–15 SC/100 muscle fibers reported in resistance-trained muscle (Petrella et al., 2008; Mackey et al., 2011; Suetta et al., 2013; Farup et al., 2014). The larger MyHC II SC pool in the present study is supported by previous training studies as both 14 weeks (Verney et al., 2008) and 10 weeks (Hoedt et al., 2016) of bicycle training led to more satellite cell accretion around MyHC II muscle fibers than MyHC I fibers. The only exception is the increase in MyHC I, but not MyHC II satellite cells noted in subjects that performed 12 weeks of aerobic cycling (Fry et al., 2014). The lack of MyHC II SC expansion may be attributed to the short duration of the training program and/or the absence of high intensity cycling, as Hoedt and colleagues speculated (Hoedt et al., 2016). The rich SC content associated with MyHC I and II fibers in the current project probably reflect the chronic bicycle training performed prior to the study, altogether illustrating the potential for skeletal muscle to support an extensive satellite cell pool in aerobically trained muscle.

The high fitness level of the current group allowed us to provide new insight into how the muscle stem cell population of trained skeletal muscle responds to a significant training insult. Both MyHC I and MyHC II fibers were sensitive to the block of intensified cycling as both fiber types possessed more satellite cells 10 days following the completion of ICT_CHO_. Interestingly, the time course of satellite cell expansion was different between fiber types such that MyHC I SC were consistently higher (8 of 8 subjects) immediately following ICT, whereas MyHC II SC's increased in only 5 of 8 subjects immediately following ICT and did not statistically differ until 10 days following the completion of ICT. The apparent difference in the SC timecourse between fiber types was also accompanied by similar temporal differences in myonuclear accretion. The number of MyHC I nuclei was elevated immediately after ICT, whereas the abundance of MyHC II nuclei was unchanged after ICT, but higher following 10 days of recovery. Though the mechanisms are unknown, the delayed myogenic response of the MyHC II fibers seems to be consistent with the general vulnerability of MHyC II fibers during periods of heavy aerobic training. For instance, 10 days of heavy swim training reduced MyHC IIa (but not MyHC I) muscle fiber size and contractile function (Fitts et al., 1989). Consistent with the idea that MyHC IIa fibers are especially sensitive to heavy training, MyHC IIa fibers undergo substantial remodeling when provided with a period of recovery (Trappe et al., 2000, 2006; Neary et al., 2003; Luden et al., 2010). More specific to the myogenic program, the induction of FN14 mRNA following running exercise was suppressed in MyHC IIa muscle fibers relative to the response elicited after 3 weeks of tapered training (Murach et al., 2014). This is relevant here because FN14 and the associated TWEAK pathway have been implicated in the regulation of myogenesis (Enwere et al., 2012, 2014).

Regardless of the time course discussed above, the increase in myonuclei was somewhat surprising. We hypothesized that an increase in satellite cell content would occur without myonuclear accretion, much like what has been observed following aerobic training studies (Charifi et al., 2003; Verney et al., 2008; Kurosaka et al., 2012; Hoedt et al., 2016). The primary difference between the current work and previous investigations is the severity of the training stimulus as the intensified training performed here was an overload stimulus that acutely impaired performance (D'Lugos et al., 2016); severe training loads may necessitate additional myonuclei to support transcriptional activity involved in muscle remodeling. This is substantiated by a recent report that myonuclear number was more than 2-fold higher in elite junior cyclists following their competitive season (Frese et al., 2016). There is contrasting evidence though as 6 weeks of high intensity interval training in previously untrained subjects increased satellite cells associated with hybrid muscle fibers (fibers possessing both MyHC I and MyHC II protein), without myonuclear accretion (Joanisse et al., 2013). However, perhaps because of the untrained status of the subjects, the fibers also showed clear signs of remodeling (specifically myoblast/myotube fusion) based on an increase in centrally located nuclei; suggesting that fate priority was myoblast fusion and not myonuclear accretion. This is different than the current findings, as we observed very few centrally located nuclei.

While there was clear evidence of a fiber-type specific SC and myonuclear response under CHO conditions, the only substantive change following ICT with PRO supplementation was an increase in the number of MyHC I satellite cells. This occurred in the absence of myonuclear changes, suggesting that the molecular cues for MyHC I satellite cell proliferation were present but that the training did not call for an increase in myonuclei. When coupled with the MyHC I (14%) and II fiber growth (16%) previously reported in these subjects (D'Lugos et al., 2016), we are led to believe that skeletal muscle better tolerated the heavy training with PRO compared to CHO. A recent report supports this belief, demonstrating that CHO+PRO supplementation proximal to acute, heavy endurance exercise to not only attenuate the acute (4 h) inflammatory transcriptional response in skeletal muscle, but also shift the transcriptome toward a muscle growth and regenerative response compared to CHO supplementation (Rowlands et al., 2016). Still, relatively little is understood of how PRO supplementation impacts the SC response to aerobic training. What is known of CHO+PRO intake around the time of aerobic exercise is that it can attenuate markers of proteolysis (Harber et al., 2010), reduce net protein breakdown (Koopman et al., 2004), increase protein synthesis and whole body protein balance (Howarth et al., 2009) and decrease markers of muscle damage compared to CHO or placebo treatments (Saunders et al., 2004; Valentine et al., 2008), altogether indicating that protein supplementation can be advantageous for skeletal muscle during periods of heavy exercise. Interestingly, there is compelling evidence that leucine provision can promote swift proliferative activity in pig myogenic progenitor cells (Han et al., 2008) and that whey protein supplementation in humans can expedite and amplify satellite cell dynamics following resistance exercise (Olsen et al., 2006; Farup et al., 2014). Therefore, when coupled with the idea that PRO supplementation was protective during ICT, it seems possible that the increase in MyHC I SC simply reflects enhanced myogenic sensitivity to exercise stress with PRO.

The value of these findings emanate from the experimental design, whereby trained skeletal muscle was exposed to a significant training perturbation. While this allowed us to document how SC and myonuclear dynamics respond to heavy exercise when already equipped with a relatively large satellite cell pool, the inclusion of direct markers of SC activation and SC fate would have considerably strengthened these data. Regardless, the current findings extend our understanding of the diverse conditions (training and nutrition) that can elicit satellite cell and myonuclear responses. The increase in SC and myonuclear content in the absence of hypertrophy (CHO) along with a limited SC and myonuclear response in the presence of cellular hypertrophy (PRO) may seem paradoxical. However, our data supports recent reports proposing alternative roles for SC incorporation into skeletal muscle, other than to accomplish myofiber hypertrophy (Joanisse et al., 2013, 2015). Further, although SC pool expansion and myonuclear accretion are typically believed to be beneficial and perhaps necessary for large-scale hypertrophy, we think it is possible that the modest hypertrophy and limited SC and myonuclear response to ICT with PRO may reflect better tolerance to the heavy block of training, particularly when coupled with our previous report that PRO enhanced skeletal muscle recovery.

## Author contributions

AM, NL, MS, and AD conception and design of research; AM, AD, NL, MS, and KG performed experiments; AM, NL, and KG analyzed data; AM, NL, interpreted results of experiments; AM and NL drafted manuscript; AM, AD, KG, NL, and MS approved final version of manuscript.

## Funding

This project was supported by James Madison University's College of Health and Behavioral Studies Faculty Research Grant (to NL and AM), as well as a grant from the Dairy Research Institute (NL and MS).

### Conflict of interest statement

MS has served as a member of advisory committees for the National Dairy Council, and the National FluidMilk Processor Promotion Board, and has received fees and travel reimbursement for work related to this role. Otherwise, no conflicts of interest, financial or otherwise, are declared by the authors. Two of the reviewers KAM and IJV and handling Editor declared their shared affiliation, and the handling Editor states that the process nevertheless met the standards of a fair and objective review.
